# 1-Methyl-4-({5-[(4-methyl­phen­yl)sulfan­yl]pent­yl}sulfan­yl)benzene

**DOI:** 10.1107/S1600536809044870

**Published:** 2009-11-04

**Authors:** Iván Brito, Alejandro Cárdenas, Joselyn Albanez, Michael Bolte, Matías López-Rodríguez

**Affiliations:** a Departamento de Química, Facultad de Ciencias Básicas, Universidad de Antofagasta, Casilla 170, Antofagasta, Chile; b Departamento de Física, Facultad de Ciencias Básicas, Universidad de Antofagasta, Casilla 170, Antofagasta, Chile; c Institut für Anorganische Chemie der Goethe-Universität Frankfurt, Max-von-Laue-Strasse 7, D-60438 Frankfurt am Main, Germany; d Instituto de Bio-Orgánica ’Antonio González’, Universidad de La Laguna, Astrofísico Francisco, Sánchez N°2, La Laguna, Tenerife, Spain

## Abstract

There are two independent mol­ecules in the asymmetric unit of the title compound, C_19_H_24_S_2_. In both mol­ecules, the aliphatic segment of the ligand is in an all-*trans* conformation: the –S–(CH_2_)_5_–S–bridging chain is almost planar (r.m.s. deviation for all non-H atoms = 0.0393 and 0.0796 Å in the two mol­ecules) and maximally extended. Their mean planes form dihedral angles of 4.08 (6)/20.47 (6) and 2.22 (6)/58.19 (6)° with the aromatic rings in the two mol­ecules. The crystal packing is purely governed by weak inter­molecular forces.

## Related literature

For the potential of coordination polymers based on multitopic bridging ligands and metal centers as functional mat­erials, see: Guo *et al.* (2002 ▶); Melcer *et al.* (2001 ▶). For the use of flexible ligands in such structures, see: Bu *et al.* (2001 ▶); Withersby *et al.* (1997 ▶). For our studies on the synthesis and structural characterization of S(II) compounds, see: Brito *et al.* (2004 ▶, 2005 ▶, 2006 ▶). For comparison bond distances in several phenylthioether compounds, see: Murray & Hartley (1981 ▶).
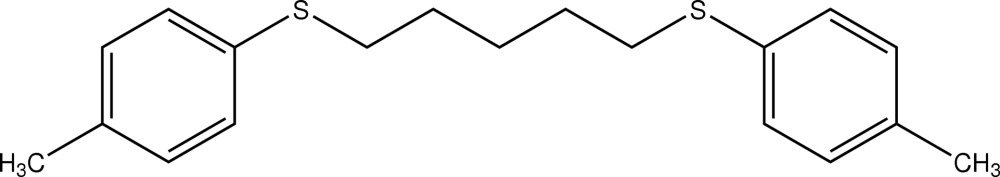


## Experimental

### 

#### Crystal data

C_19_H_24_S_2_
*M* *_r_* = 316.5
Triclinic, 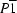

*a* = 5.7350 (3) Å
*b* = 14.8989 (7) Å
*c* = 20.5873 (9) Å
α = 84.586 (4)°
β = 88.355 (4)°
γ = 83.937 (4)°
*V* = 1741.13 (14) Å^3^
*Z* = 4
Mo *K*α radiation
μ = 0.30 mm^−1^
*T* = 173 K
0.37 × 0.32 × 0.3 mm

#### Data collection

Stoe IPDS II two-circle diffractometer
Absorption correction: multi-scan (*MULABS*; Spek, 2003 ▶; Blessing, 1995 ▶) *T*_min_ = 0.898, *T*_max_ = 0.916
31941 measured reflections
7762 independent reflections
6312 reflections with *I* > 2σ(*I*)
*R*_int_ = 0.046

#### Refinement

*R*[*F* ^2^ > 2σ(*F* ^2^)] = 0.035
*wR*(*F* ^2^) = 0.095
*S* = 1.00
7762 reflections
383 parameters
H-atom parameters constrained
Δρ_max_ = 0.17 e Å^−3^
Δρ_min_ = −0.40 e Å^−3^

### 

Data collection: *X-AREA* (Stoe & Cie, 2001 ▶); cell refinement: *X-AREA*; data reduction: *X-AREA*; program(s) used to solve structure: *SHELXS97* (Sheldrick, 2008 ▶); program(s) used to refine structure: *SHELXL97* (Sheldrick, 2008 ▶); molecular graphics: *XP* (Sheldrick, 2008 ▶); software used to prepare material for publication: *WinGX* (Farrugia, 1999 ▶).

## Supplementary Material

Crystal structure: contains datablocks global, I. DOI: 10.1107/S1600536809044870/om2292sup1.cif

Structure factors: contains datablocks I. DOI: 10.1107/S1600536809044870/om2292Isup2.hkl

Additional supplementary materials:  crystallographic information; 3D view; checkCIF report

## Figures and Tables

**Fig. 1.**
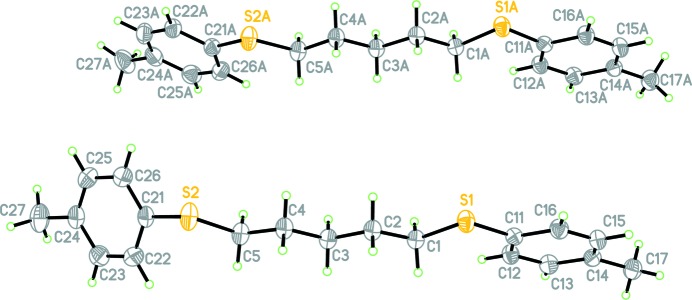
A view of the two independent molecules of the title compound, with the atom-numbering scheme (A bottom and B top). Displacement ellipsoids are drawn at the 50% probability level and H atoms are shown as small spheres of arbitrary radii.

## supplementary crystallographic information

### Comment

In recent years, the rational design of coordination polymers based on multitopic bridging ligands and metal centers represents one of the most rapidly developing fields owing to their potential as functional materials (Guo *et al.*, 2002; Melcer *et al.*, 2001). The use of flexible ligands in such studies has attracted increasing attention because the flexibility and conformational freedom of such ligands offer the possibility for the construction of diverse frameworks with tailored properties and functions (Bu *et al.*, 2001; Withersby *et al.*, 1997).

The structure of the title compound is described here as part of our work involving the study of the synthesis and structural characterization of divalent-sulfur compounds (Brito *et al.*, 2004, 2005, 2006). In both molecules the aliphatic segment of this ligand is in an all-*trans* conformation. The bridging chain moiety, –S-(CH_2_)_5_-S–, is almost planar (r.m.s. deviation for all non-H atoms: 0.0393 and 0.0796 Å). Its mean planes form a dihedral angle of 4.08 (6) and 20.47 (6)°; 2.22 (6) and 58.19 (6)° with the aromatic rings in the molecules A and B, respectively. The conformation of the central –S-(CH_2_)_5_-S– fragment is maximally extended. The average S-C*sp*^2 ^bond distance of 1.7717 (12) Å is considerably shorter than the average S-C*sp*^3 ^distance of 1.8151 (12) Å; corresponding bond distances in several phenylthioether compounds (Murray & Hartley, 1981) are 1.75 and 1.81Å respectively. The bond angles at sulfur [average 103.8 (6)°] are less than tetrahedral as is usually found in simple sulfides.

### Experimental

The title compound was synthesized as follows: A solution of 1,5-dibromopentane (1.15 g, 5 mmol) in ethanol (10 ml) was added dropwise to a mixture of p-thiocresol (1.36 g, 11 mmol), KOH (0.615 g, 11 mmol) and ethanol (10 ml). The reaction mixture was stirred for 24 h at room temperature. The precipitate was filtered off and washed with water. Yield 74%; m.p. 317 K. FT—IR (KBr pellets, cm^-1^): ν (s, C—H of CH_3_ (*asym*)) 2980, ν (w, C—H of CH_3_(*sym*)) 2847, ν (w, C—H (aliphatic chain, *sym*) 2944, ν (w, C—H (chain aliphatic, *asym*) 2924, ν (s, C—H disubstitution 1,2) 1424,, ν (w, C—S) 731. Crystals suitable for single-crystals X-ray analysis were obtained by recrystallization from an acetonitrile solution.

### Refinement

Hydrogen atoms were located in a difference Fourier map but they were included in calculated positions [C—H = 0.95 - 0.99 Å] and refined as riding [*U*_iso_(H) = 1.2*U*_eq_(C) or 1.5*U*_eq_(C_methyl_)]. The methyl groups in one molecule were refined as being disordered over two equally populated sites. All methyl groups were allowed to rotate but not to tip.

### Crystal data

**Table:**

C_19_H_24_S_2_	*Z* = 4
*M**_r_* = 316.5	*F*(000) = 680
Triclinic, *P*1	*D*_x_ = 1.207 Mg m^−^^3^
Hall symbol: -P 1	Mo *K*α radiation, λ = 0.71073 Å
*a* = 5.7350 (3) Å	Cell parameters from 28731 reflections
*b* = 14.8989 (7) Å	θ = 2.4–27.6°
*c* = 20.5873 (9) Å	µ = 0.30 mm^−^^1^
α = 84.586 (4)°	*T* = 173 K
β = 88.355 (4)°	Block, colourless
γ = 83.937 (4)°	0.37 × 0.32 × 0.3 mm
*V* = 1741.13 (14) Å^3^	

### Data collection

**Table:**

Stoe IPDS II two-circle diffractometer	6312 reflections with *I* > 2σ(*I*)
graphite	*R*_int_ = 0.046
ω scans	θ_max_ = 27.2°, θ_min_ = 2.3°
Absorption correction: multi-scan (*MULABS*; Spek, 2003; Blessing, 1995)	*h* = −7→7
*T*_min_ = 0.898, *T*_max_ = 0.916	*k* = −19→19
31941 measured reflections	*l* = −26→26
7762 independent reflections	

### Refinement

**Table:**

Refinement on *F*^2^	Primary atom site location: structure-invariant direct methods
Least-squares matrix: full	Secondary atom site location: difference Fourier map
*R*[*F*^2^ > 2σ(*F*^2^)] = 0.035	Hydrogen site location: inferred from neighbouring sites
*wR*(*F*^2^) = 0.095	H-atom parameters constrained
*S* = 1.00	*w* = 1/[σ^2^(*F*_o_^2^) + (0.0641*P*)^2^] where *P* = (*F*_o_^2^ + 2*F*_c_^2^)/3
7762 reflections	(Δ/σ)_max_ = 0.001
383 parameters	Δρ_max_ = 0.17 e Å^−^^3^
0 restraints	Δρ_min_ = −0.40 e Å^−^^3^

### Special details

**Table:**

Geometry. All e.s.d.'s (except the e.s.d. in the dihedral angle between two l.s. planes) are estimated using the full covariance matrix. The cell e.s.d.'s are taken into account individually in the estimation of e.s.d.'s in distances, angles and torsion angles; correlations between e.s.d.'s in cell parameters are only used when they are defined by crystal symmetry. An approximate (isotropic) treatment of cell e.s.d.'s is used for estimating e.s.d.'s involving l.s. planes.
Refinement. Refinement of *F*^2^ against ALL reflections. The weighted *R*-factor *wR* and goodness of fit *S* are based on *F*^2^, conventional *R*-factors *R* are based on *F*, with *F* set to zero for negative *F*^2^. The threshold expression of *F*^2^ > σ(*F*^2^) is used only for calculating *R*-factors(gt) *etc*. and is not relevant to the choice of reflections for refinement. *R*-factors based on *F*^2^ are statistically about twice as large as those based on *F*, and *R*- factors based on ALL data will be even larger.

### Fractional atomic coordinates and isotropic or equivalent isotropic displacement parameters (Å^2^)

**Table:**

	*x*	*y*	*z*	*U*_iso_*/*U*_eq_	Occ. (<1)
S1	0.47078 (5)	0.34976 (2)	0.553712 (14)	0.03300 (8)	
S2	0.28810 (6)	0.27344 (2)	0.186815 (15)	0.04058 (9)	
C1	0.6130 (2)	0.37163 (8)	0.47493 (5)	0.0295 (2)	
H1A	0.6077	0.4377	0.4626	0.035*	
H1B	0.7791	0.3455	0.4761	0.035*	
C2	0.4783 (2)	0.32682 (8)	0.42613 (5)	0.0308 (2)	
H2A	0.4963	0.2604	0.4375	0.037*	
H2B	0.3094	0.3482	0.4299	0.037*	
C3	0.5604 (2)	0.34685 (8)	0.35559 (6)	0.0311 (2)	
H3A	0.7281	0.3242	0.3511	0.037*	
H3B	0.5444	0.4132	0.344	0.037*	
C4	0.4164 (2)	0.30178 (8)	0.30902 (6)	0.0321 (2)	
H4A	0.4401	0.2352	0.3193	0.039*	
H4B	0.2479	0.3215	0.3159	0.039*	
C5	0.4831 (2)	0.32489 (9)	0.23764 (6)	0.0348 (3)	
H5A	0.648	0.3013	0.2293	0.042*	
H5B	0.4674	0.3915	0.2272	0.042*	
C11	0.64268 (19)	0.39437 (7)	0.61017 (5)	0.0271 (2)	
C12	0.8360 (2)	0.44212 (8)	0.59405 (6)	0.0310 (2)	
H12	0.8835	0.4529	0.5497	0.037*	
C13	0.9589 (2)	0.47391 (8)	0.64276 (6)	0.0325 (2)	
H13	1.0902	0.5063	0.631	0.039*	
C14	0.8954 (2)	0.45972 (8)	0.70837 (6)	0.0305 (2)	
C15	0.7008 (2)	0.41199 (8)	0.72387 (6)	0.0324 (2)	
H15	0.6531	0.4015	0.7683	0.039*	
C16	0.5764 (2)	0.37974 (8)	0.67601 (6)	0.0317 (2)	
H16	0.4451	0.3474	0.6879	0.038*	
C17	1.0314 (2)	0.49382 (9)	0.76097 (6)	0.0399 (3)	
H17A	1.1982	0.4899	0.7486	0.06*	0.5
H17B	1.0107	0.4567	0.8022	0.06*	0.5
H17C	0.9737	0.5571	0.7663	0.06*	0.5
H17D	0.9235	0.5125	0.7961	0.06*	0.5
H17E	1.1111	0.5457	0.7425	0.06*	0.5
H17F	1.148	0.4454	0.7784	0.06*	0.5
C21	0.3592 (2)	0.32102 (8)	0.10740 (6)	0.0319 (2)	
C22	0.5809 (2)	0.30352 (9)	0.07944 (6)	0.0393 (3)	
H22	0.7016	0.269	0.1044	0.047*	
C23	0.6276 (2)	0.33592 (9)	0.01566 (6)	0.0412 (3)	
H23	0.7806	0.3236	−0.0024	0.049*	
C24	0.4553 (2)	0.38607 (8)	−0.02229 (6)	0.0374 (3)	
C25	0.2359 (2)	0.40369 (9)	0.00603 (6)	0.0435 (3)	
H25	0.115	0.4378	−0.0191	0.052*	
C26	0.1878 (2)	0.37279 (9)	0.07023 (6)	0.0404 (3)	
H26	0.0365	0.3872	0.0888	0.048*	
C27	0.5064 (3)	0.41786 (11)	−0.09241 (7)	0.0546 (4)	
H27A	0.6694	0.4321	−0.097	0.082*	0.5
H27B	0.4009	0.4722	−0.1056	0.082*	0.5
H27C	0.4822	0.3699	−0.1203	0.082*	0.5
H27D	0.3656	0.4174	−0.1182	0.082*	0.5
H27E	0.6341	0.3773	−0.1097	0.082*	0.5
H27F	0.5528	0.4796	−0.095	0.082*	0.5
S1A	−0.03887 (5)	0.84904 (2)	0.557886 (14)	0.03438 (9)	
S2A	−0.12726 (6)	0.76554 (2)	0.184136 (15)	0.04023 (9)	
C1A	0.1026 (2)	0.87023 (8)	0.47906 (5)	0.0304 (2)	
H1A1	0.098	0.9362	0.4668	0.037*	
H1A2	0.2685	0.844	0.4803	0.037*	
C2A	−0.0309 (2)	0.82581 (8)	0.42979 (6)	0.0314 (2)	
H2A1	−0.017	0.7595	0.4414	0.038*	
H2A2	−0.1992	0.8486	0.4321	0.038*	
C3A	0.0611 (2)	0.84496 (8)	0.36032 (6)	0.0320 (2)	
H3A1	0.2301	0.8232	0.3582	0.038*	
H3A2	0.0446	0.9112	0.3484	0.038*	
C4A	−0.0691 (2)	0.79927 (8)	0.31102 (6)	0.0330 (2)	
H4A1	−0.0531	0.7329	0.3226	0.04*	
H4A2	−0.238	0.8213	0.3125	0.04*	
C5A	0.0293 (2)	0.81981 (8)	0.24225 (6)	0.0348 (3)	
H5A1	0.198	0.7975	0.2406	0.042*	
H5A2	0.0137	0.8861	0.2306	0.042*	
C11A	0.13591 (19)	0.89409 (7)	0.61353 (5)	0.0280 (2)	
C12A	0.3278 (2)	0.94183 (8)	0.59637 (6)	0.0320 (2)	
H12A	0.3739	0.9519	0.5518	0.038*	
C13A	0.4519 (2)	0.97471 (8)	0.64446 (6)	0.0337 (3)	
H13A	0.5825	1.0072	0.632	0.04*	
C14A	0.3910 (2)	0.96155 (8)	0.71021 (6)	0.0321 (2)	
C15A	0.1984 (2)	0.91322 (8)	0.72665 (6)	0.0349 (3)	
H15A	0.1526	0.9031	0.7712	0.042*	
C16A	0.0728 (2)	0.87984 (8)	0.67958 (6)	0.0335 (3)	
H16A	−0.0571	0.847	0.6921	0.04*	
C17A	0.5269 (2)	0.99751 (10)	0.76193 (7)	0.0422 (3)	
H17G	0.66	1.0263	0.7415	0.063*	
H17H	0.5846	0.9474	0.7936	0.063*	
H17I	0.4243	1.0422	0.7843	0.063*	
C21A	−0.0231 (2)	0.81398 (8)	0.10834 (6)	0.0330 (2)	
C22A	−0.1589 (2)	0.81022 (8)	0.05391 (6)	0.0380 (3)	
H22A	−0.2998	0.7818	0.0585	0.046*	
C23A	−0.0901 (3)	0.84768 (9)	−0.00686 (6)	0.0442 (3)	
H23A	−0.1842	0.8438	−0.0436	0.053*	
C24A	0.1144 (3)	0.89095 (9)	−0.01533 (6)	0.0428 (3)	
C25A	0.2483 (2)	0.89386 (9)	0.03914 (7)	0.0433 (3)	
H25A	0.3883	0.9229	0.0345	0.052*	
C26A	0.1841 (2)	0.85568 (9)	0.10038 (6)	0.0399 (3)	
H26A	0.281	0.8579	0.1367	0.048*	
C27A	0.1903 (3)	0.93199 (11)	−0.08128 (7)	0.0590 (4)	
H27G	0.0689	0.928	−0.1131	0.088*	
H27H	0.3375	0.8989	−0.095	0.088*	
H27I	0.2138	0.9957	−0.0786	0.088*	

### Atomic displacement parameters (Å^2^)

**Table:**

	*U*^11^	*U*^22^	*U*^33^	*U*^12^	*U*^13^	*U*^23^
S1	0.03288 (15)	0.04141 (17)	0.02700 (15)	−0.01181 (12)	0.00068 (11)	−0.00653 (12)
S2	0.0536 (2)	0.04460 (19)	0.02717 (16)	−0.02117 (14)	−0.00305 (13)	−0.00360 (13)
C1	0.0319 (5)	0.0308 (6)	0.0261 (5)	−0.0043 (4)	−0.0016 (4)	−0.0031 (4)
C2	0.0353 (6)	0.0306 (6)	0.0270 (6)	−0.0059 (4)	−0.0023 (5)	−0.0024 (4)
C3	0.0359 (6)	0.0309 (6)	0.0271 (6)	−0.0059 (5)	−0.0025 (5)	−0.0023 (4)
C4	0.0385 (6)	0.0319 (6)	0.0270 (6)	−0.0075 (5)	−0.0025 (5)	−0.0035 (5)
C5	0.0407 (6)	0.0383 (6)	0.0270 (6)	−0.0098 (5)	−0.0030 (5)	−0.0042 (5)
C11	0.0280 (5)	0.0274 (5)	0.0258 (5)	−0.0014 (4)	−0.0005 (4)	−0.0038 (4)
C12	0.0325 (6)	0.0344 (6)	0.0265 (6)	−0.0063 (5)	0.0019 (4)	−0.0029 (4)
C13	0.0307 (6)	0.0344 (6)	0.0334 (6)	−0.0081 (5)	0.0003 (5)	−0.0035 (5)
C14	0.0312 (6)	0.0304 (6)	0.0298 (6)	0.0009 (4)	−0.0035 (4)	−0.0058 (4)
C15	0.0351 (6)	0.0369 (6)	0.0248 (5)	−0.0015 (5)	0.0012 (4)	−0.0035 (5)
C16	0.0309 (6)	0.0351 (6)	0.0292 (6)	−0.0057 (4)	0.0023 (4)	−0.0018 (5)
C17	0.0397 (7)	0.0469 (7)	0.0348 (7)	−0.0040 (5)	−0.0071 (5)	−0.0118 (5)
C21	0.0396 (6)	0.0317 (6)	0.0260 (6)	−0.0071 (5)	−0.0036 (5)	−0.0062 (4)
C22	0.0374 (6)	0.0430 (7)	0.0358 (7)	0.0036 (5)	−0.0049 (5)	−0.0021 (5)
C23	0.0378 (7)	0.0493 (7)	0.0362 (7)	−0.0030 (5)	0.0025 (5)	−0.0061 (6)
C24	0.0496 (7)	0.0356 (6)	0.0290 (6)	−0.0107 (5)	−0.0047 (5)	−0.0050 (5)
C25	0.0450 (7)	0.0488 (8)	0.0350 (7)	0.0032 (6)	−0.0118 (5)	−0.0011 (6)
C26	0.0354 (6)	0.0504 (7)	0.0353 (7)	0.0002 (5)	−0.0024 (5)	−0.0080 (6)
C27	0.0749 (11)	0.0600 (9)	0.0313 (7)	−0.0205 (8)	−0.0014 (7)	−0.0010 (6)
S1A	0.03389 (16)	0.04282 (17)	0.02890 (16)	−0.01300 (12)	0.00289 (11)	−0.00710 (12)
S2A	0.05185 (19)	0.04138 (18)	0.03061 (17)	−0.01794 (14)	−0.00394 (13)	−0.00413 (13)
C1A	0.0326 (6)	0.0312 (6)	0.0278 (6)	−0.0038 (4)	0.0004 (4)	−0.0036 (4)
C2A	0.0356 (6)	0.0299 (6)	0.0292 (6)	−0.0056 (5)	−0.0010 (5)	−0.0024 (5)
C3A	0.0372 (6)	0.0307 (6)	0.0287 (6)	−0.0064 (5)	−0.0011 (5)	−0.0030 (5)
C4A	0.0396 (6)	0.0310 (6)	0.0292 (6)	−0.0061 (5)	−0.0026 (5)	−0.0034 (5)
C5A	0.0421 (7)	0.0341 (6)	0.0295 (6)	−0.0083 (5)	−0.0042 (5)	−0.0033 (5)
C11A	0.0285 (5)	0.0272 (5)	0.0285 (6)	−0.0019 (4)	0.0009 (4)	−0.0038 (4)
C12A	0.0330 (6)	0.0349 (6)	0.0284 (6)	−0.0068 (5)	0.0041 (5)	−0.0025 (5)
C13A	0.0314 (6)	0.0358 (6)	0.0351 (6)	−0.0086 (5)	0.0026 (5)	−0.0048 (5)
C14A	0.0331 (6)	0.0319 (6)	0.0317 (6)	−0.0025 (5)	−0.0014 (5)	−0.0060 (5)
C15A	0.0384 (6)	0.0394 (6)	0.0272 (6)	−0.0063 (5)	0.0030 (5)	−0.0032 (5)
C16A	0.0339 (6)	0.0371 (6)	0.0303 (6)	−0.0092 (5)	0.0038 (5)	−0.0024 (5)
C17A	0.0423 (7)	0.0496 (8)	0.0372 (7)	−0.0100 (6)	−0.0038 (6)	−0.0106 (6)
C21A	0.0376 (6)	0.0307 (6)	0.0303 (6)	0.0003 (5)	−0.0027 (5)	−0.0046 (5)
C22A	0.0408 (7)	0.0371 (6)	0.0371 (7)	−0.0014 (5)	−0.0063 (5)	−0.0097 (5)
C23A	0.0573 (8)	0.0424 (7)	0.0324 (7)	0.0040 (6)	−0.0104 (6)	−0.0081 (5)
C24A	0.0546 (8)	0.0380 (7)	0.0330 (6)	0.0066 (6)	0.0039 (6)	−0.0035 (5)
C25A	0.0418 (7)	0.0458 (7)	0.0413 (7)	−0.0029 (6)	0.0039 (6)	−0.0016 (6)
C26A	0.0377 (6)	0.0463 (7)	0.0354 (7)	−0.0034 (5)	−0.0051 (5)	−0.0024 (5)
C27A	0.0834 (12)	0.0535 (9)	0.0357 (8)	0.0062 (8)	0.0123 (7)	−0.0004 (6)

### Geometric parameters (Å, °)

**Table:**

S1—C11	1.7648 (12)	C27—H27D	0.98
S1—C1	1.8119 (12)	C27—H27E	0.98
S2—C21	1.7737 (12)	C27—H27F	0.98
S2—C5	1.8254 (12)	S1A—C11A	1.7665 (12)
C1—C2	1.5274 (16)	S1A—C1A	1.8091 (12)
C1—H1A	0.99	S2A—C21A	1.7714 (12)
C1—H1B	0.99	S2A—C5A	1.8136 (12)
C2—C3	1.5259 (16)	C1A—C2A	1.5268 (16)
C2—H2A	0.99	C1A—H1A1	0.99
C2—H2B	0.99	C1A—H1A2	0.99
C3—C4	1.5285 (16)	C2A—C3A	1.5223 (16)
C3—H3A	0.99	C2A—H2A1	0.99
C3—H3B	0.99	C2A—H2A2	0.99
C4—C5	1.5246 (16)	C3A—C4A	1.5270 (16)
C4—H4A	0.99	C3A—H3A1	0.99
C4—H4B	0.99	C3A—H3A2	0.99
C5—H5A	0.99	C4A—C5A	1.5249 (16)
C5—H5B	0.99	C4A—H4A1	0.99
C11—C12	1.3950 (16)	C4A—H4A2	0.99
C11—C16	1.4006 (15)	C5A—H5A1	0.99
C12—C13	1.3886 (17)	C5A—H5A2	0.99
C12—H12	0.95	C11A—C12A	1.3917 (16)
C13—C14	1.3914 (16)	C11A—C16A	1.3995 (16)
C13—H13	0.95	C12A—C13A	1.3904 (17)
C14—C15	1.3989 (17)	C12A—H12A	0.95
C14—C17	1.5083 (16)	C13A—C14A	1.3893 (17)
C15—C16	1.3829 (17)	C13A—H13A	0.95
C15—H15	0.95	C14A—C15A	1.3979 (17)
C16—H16	0.95	C14A—C17A	1.5071 (17)
C17—H17A	0.98	C15A—C16A	1.3826 (17)
C17—H17B	0.98	C15A—H15A	0.95
C17—H17C	0.98	C16A—H16A	0.95
C17—H17D	0.98	C17A—H17G	0.98
C17—H17E	0.98	C17A—H17H	0.98
C17—H17F	0.98	C17A—H17I	0.98
C21—C26	1.3850 (17)	C21A—C22A	1.3917 (17)
C21—C22	1.3898 (17)	C21A—C26A	1.3966 (18)
C22—C23	1.3843 (18)	C22A—C23A	1.3853 (19)
C22—H22	0.95	C22A—H22A	0.95
C23—C24	1.3871 (19)	C23A—C24A	1.396 (2)
C23—H23	0.95	C23A—H23A	0.95
C24—C25	1.3816 (19)	C24A—C25A	1.384 (2)
C24—C27	1.5069 (18)	C24A—C27A	1.5073 (19)
C25—C26	1.3878 (18)	C25A—C26A	1.3895 (18)
C25—H25	0.95	C25A—H25A	0.95
C26—H26	0.95	C26A—H26A	0.95
C27—H27A	0.98	C27A—H27G	0.98
C27—H27B	0.98	C27A—H27H	0.98
C27—H27C	0.98	C27A—H27I	0.98
			
C11—S1—C1	105.56 (5)	C24—C27—H27D	109.5
C21—S2—C5	102.19 (6)	H27A—C27—H27D	141.1
C2—C1—S1	106.39 (8)	H27B—C27—H27D	56.3
C2—C1—H1A	110.5	H27C—C27—H27D	56.3
S1—C1—H1A	110.5	C24—C27—H27E	109.5
C2—C1—H1B	110.5	H27A—C27—H27E	56.3
S1—C1—H1B	110.5	H27B—C27—H27E	141.1
H1A—C1—H1B	108.6	H27C—C27—H27E	56.3
C3—C2—C1	113.25 (9)	H27D—C27—H27E	109.5
C3—C2—H2A	108.9	C24—C27—H27F	109.5
C1—C2—H2A	108.9	H27A—C27—H27F	56.3
C3—C2—H2B	108.9	H27B—C27—H27F	56.3
C1—C2—H2B	108.9	H27C—C27—H27F	141.1
H2A—C2—H2B	107.7	H27D—C27—H27F	109.5
C2—C3—C4	110.84 (10)	H27E—C27—H27F	109.5
C2—C3—H3A	109.5	C11A—S1A—C1A	105.09 (5)
C4—C3—H3A	109.5	C21A—S2A—C5A	102.30 (6)
C2—C3—H3B	109.5	C2A—C1A—S1A	107.21 (8)
C4—C3—H3B	109.5	C2A—C1A—H1A1	110.3
H3A—C3—H3B	108.1	S1A—C1A—H1A1	110.3
C5—C4—C3	112.47 (10)	C2A—C1A—H1A2	110.3
C5—C4—H4A	109.1	S1A—C1A—H1A2	110.3
C3—C4—H4A	109.1	H1A1—C1A—H1A2	108.5
C5—C4—H4B	109.1	C3A—C2A—C1A	112.15 (10)
C3—C4—H4B	109.1	C3A—C2A—H2A1	109.2
H4A—C4—H4B	107.8	C1A—C2A—H2A1	109.2
C4—C5—S2	108.48 (8)	C3A—C2A—H2A2	109.2
C4—C5—H5A	110	C1A—C2A—H2A2	109.2
S2—C5—H5A	110	H2A1—C2A—H2A2	107.9
C4—C5—H5B	110	C2A—C3A—C4A	112.32 (10)
S2—C5—H5B	110	C2A—C3A—H3A1	109.1
H5A—C5—H5B	108.4	C4A—C3A—H3A1	109.1
C12—C11—C16	118.61 (10)	C2A—C3A—H3A2	109.1
C12—C11—S1	125.13 (9)	C4A—C3A—H3A2	109.1
C16—C11—S1	116.26 (9)	H3A1—C3A—H3A2	107.9
C13—C12—C11	120.12 (10)	C5A—C4A—C3A	110.57 (10)
C13—C12—H12	119.9	C5A—C4A—H4A1	109.5
C11—C12—H12	119.9	C3A—C4A—H4A1	109.5
C12—C13—C14	121.89 (11)	C5A—C4A—H4A2	109.5
C12—C13—H13	119.1	C3A—C4A—H4A2	109.5
C14—C13—H13	119.1	H4A1—C4A—H4A2	108.1
C13—C14—C15	117.42 (11)	C4A—C5A—S2A	110.17 (8)
C13—C14—C17	121.49 (11)	C4A—C5A—H5A1	109.6
C15—C14—C17	121.09 (11)	S2A—C5A—H5A1	109.6
C16—C15—C14	121.49 (11)	C4A—C5A—H5A2	109.6
C16—C15—H15	119.3	S2A—C5A—H5A2	109.6
C14—C15—H15	119.3	H5A1—C5A—H5A2	108.1
C15—C16—C11	120.47 (11)	C12A—C11A—C16A	118.75 (11)
C15—C16—H16	119.8	C12A—C11A—S1A	124.98 (9)
C11—C16—H16	119.8	C16A—C11A—S1A	116.28 (9)
C14—C17—H17A	109.5	C13A—C12A—C11A	119.97 (11)
C14—C17—H17B	109.5	C13A—C12A—H12A	120
H17A—C17—H17B	109.5	C11A—C12A—H12A	120
C14—C17—H17C	109.5	C14A—C13A—C12A	122.03 (11)
H17A—C17—H17C	109.5	C14A—C13A—H13A	119
H17B—C17—H17C	109.5	C12A—C13A—H13A	119
C14—C17—H17D	109.5	C13A—C14A—C15A	117.29 (11)
H17A—C17—H17D	141.1	C13A—C14A—C17A	121.57 (11)
H17B—C17—H17D	56.3	C15A—C14A—C17A	121.14 (11)
H17C—C17—H17D	56.3	C16A—C15A—C14A	121.59 (11)
C14—C17—H17E	109.5	C16A—C15A—H15A	119.2
H17A—C17—H17E	56.3	C14A—C15A—H15A	119.2
H17B—C17—H17E	141.1	C15A—C16A—C11A	120.38 (11)
H17C—C17—H17E	56.3	C15A—C16A—H16A	119.8
H17D—C17—H17E	109.5	C11A—C16A—H16A	119.8
C14—C17—H17F	109.5	C14A—C17A—H17G	109.5
H17A—C17—H17F	56.3	C14A—C17A—H17H	109.5
H17B—C17—H17F	56.3	H17G—C17A—H17H	109.5
H17C—C17—H17F	141.1	C14A—C17A—H17I	109.5
H17D—C17—H17F	109.5	H17G—C17A—H17I	109.5
H17E—C17—H17F	109.5	H17H—C17A—H17I	109.5
C26—C21—C22	118.36 (11)	C22A—C21A—C26A	118.74 (12)
C26—C21—S2	119.88 (10)	C22A—C21A—S2A	117.21 (10)
C22—C21—S2	121.66 (9)	C26A—C21A—S2A	124.05 (10)
C23—C22—C21	120.69 (11)	C23A—C22A—C21A	120.49 (13)
C23—C22—H22	119.7	C23A—C22A—H22A	119.8
C21—C22—H22	119.7	C21A—C22A—H22A	119.8
C22—C23—C24	121.22 (12)	C22A—C23A—C24A	121.39 (13)
C22—C23—H23	119.4	C22A—C23A—H23A	119.3
C24—C23—H23	119.4	C24A—C23A—H23A	119.3
C25—C24—C23	117.71 (11)	C25A—C24A—C23A	117.50 (12)
C25—C24—C27	121.70 (12)	C25A—C24A—C27A	120.79 (14)
C23—C24—C27	120.58 (13)	C23A—C24A—C27A	121.71 (14)
C24—C25—C26	121.62 (12)	C24A—C25A—C26A	122.06 (13)
C24—C25—H25	119.2	C24A—C25A—H25A	119
C26—C25—H25	119.2	C26A—C25A—H25A	119
C21—C26—C25	120.38 (12)	C25A—C26A—C21A	119.82 (12)
C21—C26—H26	119.8	C25A—C26A—H26A	120.1
C25—C26—H26	119.8	C21A—C26A—H26A	120.1
C24—C27—H27A	109.5	C24A—C27A—H27G	109.5
C24—C27—H27B	109.5	C24A—C27A—H27H	109.5
H27A—C27—H27B	109.5	H27G—C27A—H27H	109.5
C24—C27—H27C	109.5	C24A—C27A—H27I	109.5
H27A—C27—H27C	109.5	H27G—C27A—H27I	109.5
H27B—C27—H27C	109.5	H27H—C27A—H27I	109.5
			
C11—S1—C1—C2	175.56 (7)	C11A—S1A—C1A—C2A	175.59 (8)
S1—C1—C2—C3	174.21 (8)	S1A—C1A—C2A—C3A	175.80 (8)
C1—C2—C3—C4	−178.93 (9)	C1A—C2A—C3A—C4A	178.99 (10)
C2—C3—C4—C5	176.52 (10)	C2A—C3A—C4A—C5A	−179.72 (10)
C3—C4—C5—S2	−176.47 (8)	C3A—C4A—C5A—S2A	−179.81 (8)
C21—S2—C5—C4	171.38 (8)	C21A—S2A—C5A—C4A	170.04 (8)
C1—S1—C11—C12	4.37 (12)	C1A—S1A—C11A—C12A	4.87 (12)
C1—S1—C11—C16	−175.58 (8)	C1A—S1A—C11A—C16A	−175.20 (9)
C16—C11—C12—C13	0.17 (17)	C16A—C11A—C12A—C13A	−0.31 (17)
S1—C11—C12—C13	−179.79 (9)	S1A—C11A—C12A—C13A	179.62 (9)
C11—C12—C13—C14	−0.04 (18)	C11A—C12A—C13A—C14A	0.00 (19)
C12—C13—C14—C15	−0.15 (17)	C12A—C13A—C14A—C15A	0.20 (18)
C12—C13—C14—C17	179.46 (11)	C12A—C13A—C14A—C17A	−179.99 (11)
C13—C14—C15—C16	0.23 (17)	C13A—C14A—C15A—C16A	−0.09 (18)
C17—C14—C15—C16	−179.39 (11)	C17A—C14A—C15A—C16A	−179.90 (12)
C14—C15—C16—C11	−0.10 (18)	C14A—C15A—C16A—C11A	−0.22 (19)
C12—C11—C16—C15	−0.10 (17)	C12A—C11A—C16A—C15A	0.41 (18)
S1—C11—C16—C15	179.86 (9)	S1A—C11A—C16A—C15A	−179.52 (9)
C5—S2—C21—C26	−119.79 (11)	C5A—S2A—C21A—C22A	−159.84 (10)
C5—S2—C21—C22	64.01 (11)	C5A—S2A—C21A—C26A	19.71 (12)
C26—C21—C22—C23	−1.01 (19)	C26A—C21A—C22A—C23A	−0.38 (18)
S2—C21—C22—C23	175.24 (10)	S2A—C21A—C22A—C23A	179.19 (10)
C21—C22—C23—C24	−0.4 (2)	C21A—C22A—C23A—C24A	−0.73 (19)
C22—C23—C24—C25	0.9 (2)	C22A—C23A—C24A—C25A	0.97 (19)
C22—C23—C24—C27	−177.69 (13)	C22A—C23A—C24A—C27A	−179.92 (12)
C23—C24—C25—C26	0.1 (2)	C23A—C24A—C25A—C26A	−0.1 (2)
C27—C24—C25—C26	178.68 (13)	C27A—C24A—C25A—C26A	−179.23 (13)
C22—C21—C26—C25	2.00 (19)	C24A—C25A—C26A—C21A	−1.0 (2)
S2—C21—C26—C25	−174.33 (10)	C22A—C21A—C26A—C25A	1.22 (19)
C24—C25—C26—C21	−1.6 (2)	S2A—C21A—C26A—C25A	−178.33 (10)
